# Metabolic characteristics and chemical basis of matcha from cultivars with different leaf colors

**DOI:** 10.3389/fnut.2026.1951952

**Published:** 2026-10-19

**Authors:** Shiqi Ding, Dingding Liu, Haoran Liu, Yang Gong, Piao Mei, Yuanyuan Ye, Peixin Wang, Chenyu Zhang, Jiedan Chen, Chunlei Ma, Xuecheng Zhao

**Affiliations:** National Key Laboratory for Tea Plant Germplasm Innovation and Resource Utilization, Tea Research Institute of the Chinese Academy of Agricultural Sciences, Hangzhou, China

**Keywords:** aroma, flavor, leaf color, matcha, metabolites

## Abstract

Matcha is widely used in the food, health, and cosmetic industries due to its high nutritional value and distinctive sensory properties. However, traditional matcha products are predominantly produced from green-leaf tea cultivars, which greatly limits product diversity. This study systematically compared the sensory attributes, metabolite composition, and volatile metabolite profiles of matcha produced from green-, purple-, and yellow-leaf tea cultivars to explore the biochemical basis of flavor diversity. Principal component analysis of metabolite profiles clearly separated the samples according to leaf color phenotypes, confirming a strong chemical basis for sensory differentiation among the studied cultivar groups. Green matcha was characterized by higher levels of umami amino acids and lower contents of catechins and caffeine, resulting in a fresh taste with reduced bitterness and astringency. Purple matcha showed elevated levels of ester-type catechins, caffeine, and anthocyanin-related flavonoids, leading to pronounced astringency and lingering bitterness. Yellow matcha contained the highest levels of total amino acids and theanine, contributing to a mellow taste. KEGG pathway analysis revealed that flavonoid biosynthesis and amino acid metabolism are the major pathways associated with metabolic divergence among these cultivars. Volatile analysis indicated that dihydroactinidiolide and leaf alcohol derivatives were predominant volatile compounds potentially associated with the characteristic aroma profiles. These findings provide a biochemical foundation for developing novel matcha products based on cultivars displaying distinct leaf coloration.

## 1 Introduction

Matcha is an ultra-fine powdered tea produced by steam fixation of shade-grown tea leaves, followed by drying to obtain tencha and subsequent grinding into a fine powder (1). Because of its vivid green color, distinctive aroma, complex flavor profile, and smooth mouthfeel, matcha is widely consumed and increasingly applied in foods, beverages, and functional products.

Rising consumer interest in health-promoting foods has driven growth in global demand for matcha, particularly in ready-to-drink beverages, baked goods, desserts, and nutraceutical formulations (2, 3). Considerable progress has been made in understanding the health benefits and processing characteristics of matcha. Rich in catechins, it exhibits strong antioxidant activity (4) and contributes to metabolic regulation, including modulation of the gut microbiota (5). Matcha consumption can also alleviate stress, enhance attention and memory, and, when combined with a high-fat diet, mitigate weight gain, improve glucose and lipid metabolism, reduce inflammatory cytokines, and influence the proliferation and viability of breast cancer cells (6). Regular intake of matcha is also associated with both physical and mental health benefits (7, 8).

Advances in processing technology have clarified how grinding methods and conditions influence matcha quality. Different milling techniques, including cyclone, bead, and stone milling, result in distinct particle sizes and surface morphologies, which affect the release and stability of sugars, organic acids, caffeine, and amino acids. Cyclone-milled matcha, characterized by the smallest particle size, exhibits the highest levels of these compounds and distinctive volatile profiles (9). Extended grinding time reduces particle size, enhances greenness, preserves bioactive compounds, and promotes the formation of characteristic flavor compounds such as benzaldehyde and α-ionone (10). Studies integrating sensory evaluation with non-volatile and volatile analyses have identified flavonol glycosides, aldehydes, ketones, esters, alcohols, and terpenoids to be key contributors to taste and aroma characteristics across cultivars and production regions (11, 12). Large-scale metabolomic analyses have also revealed extensive metabolic diversity in matcha, with differential metabolites enriched in pathways related to flavonoid biosynthesis, amino acid metabolism, purine metabolism, and secondary metabolite biosynthesis (13).

Most studies and commercial production have focused on green-leaf tea cultivars. However, China's tea industry has experienced rapid cultivar diversification. According to data released by the Ministry of Agriculture and Rural Affairs of China on the national seed industry big data platform, more than 400 new tea cultivars have been applied for registration over the past 5 years, ~25% of which are leaf color-variant cultivars. Representative examples include the anthocyanin-rich purple-leaf cultivar “*Zijuan*” and the yellow-leaf cultivar “*Huangjinya*,” both of which have unique biochemical profiles and high application potential. Metabolomic analysis further showed that anthocyanins and related metabolites accumulate predominantly in purple-leaf cultivars, establishing a biochemical link between leaf color and tea quality (14). Despite the availability of colored-leaf cultivars, their use in matcha production has been limited. This restricts product diversity and underutilizes valuable genetic resources. To address this gap, we systematically investigate the sensory attributes, metabolite composition, and volatile metabolite profiles of matcha produced from green-, purple-, and yellow-leaf tea cultivars. We use ultra-performance liquid chromatography-tandem mass spectrometry (UPLC-MS/MS) and gas chromatography-mass spectrometry (GC-MS) to elucidate the biochemical basis underlying differences in color, taste, and aroma among cultivars representing different leaf-color types. Our objective is to provide a scientific foundation for improving matcha quality and expanding the range of tea cultivars suitable for high-quality matcha production.

## 2 Materials and methods

### 2.1 Plant materials

Two green-leaf tea cultivars [Japanese cultivar “*Yabukita*” (G1) and Chinese cultivar “*Pingshui Rizhu*” (G2)], two purple-leaf strains (P1 and P2; hybrid progenies of the tea cultivar “*Zijuan*”) and two yellow-leaf strains (Y1 and Y2; hybrid progenies of “*Baijiguan*”) were preserved in the Shengzhou Comprehensive Experimental Field of Tea Research Institute, Chinese Academy of Agricultural Sciences. All plant materials were cultivated under identical fertilization and management conditions to minimize environmental variation and enable assessment of cultivar-dependent differences in matcha quality.

In spring 2024, tea plants of each cultivar were subjected to short-term shading prior to harvest using a black shade net providing 80% shading. The shading duration was determined according to the phenological development of each cultivar, from the fish-leaf stage to the one bud and two leaves (Supplementary Table 1). Following the shading treatment, fresh shoots consisting of one bud and two leaves were harvested from multiple plants of each cultivar grown in the same experimental field, pooled, and thoroughly mixed to form one homogeneous composite sample per cultivar. Each composite sample was then evenly divided into three portions, and each portion was immediately and independently processed into tencha under standardized conditions using an automated processing system (Anyi Intelligent Machinery Co., Ltd., Shaoxing, China). After stem removal, the leaves were steam-fixed at 100 °C for 20 s and subsequently transferred to a tencha drying furnace for multi-stage drying. During the high-temperature drying stage, the temperature reached approximately 180 °C for 3 min to facilitate rapid moisture removal and promote the development of the characteristic tencha fire aroma. The leaves were subsequently dried at 90 °C for 30 min to further reduce and stabilize the moisture content (Figure 1A).

**Figure 1:**
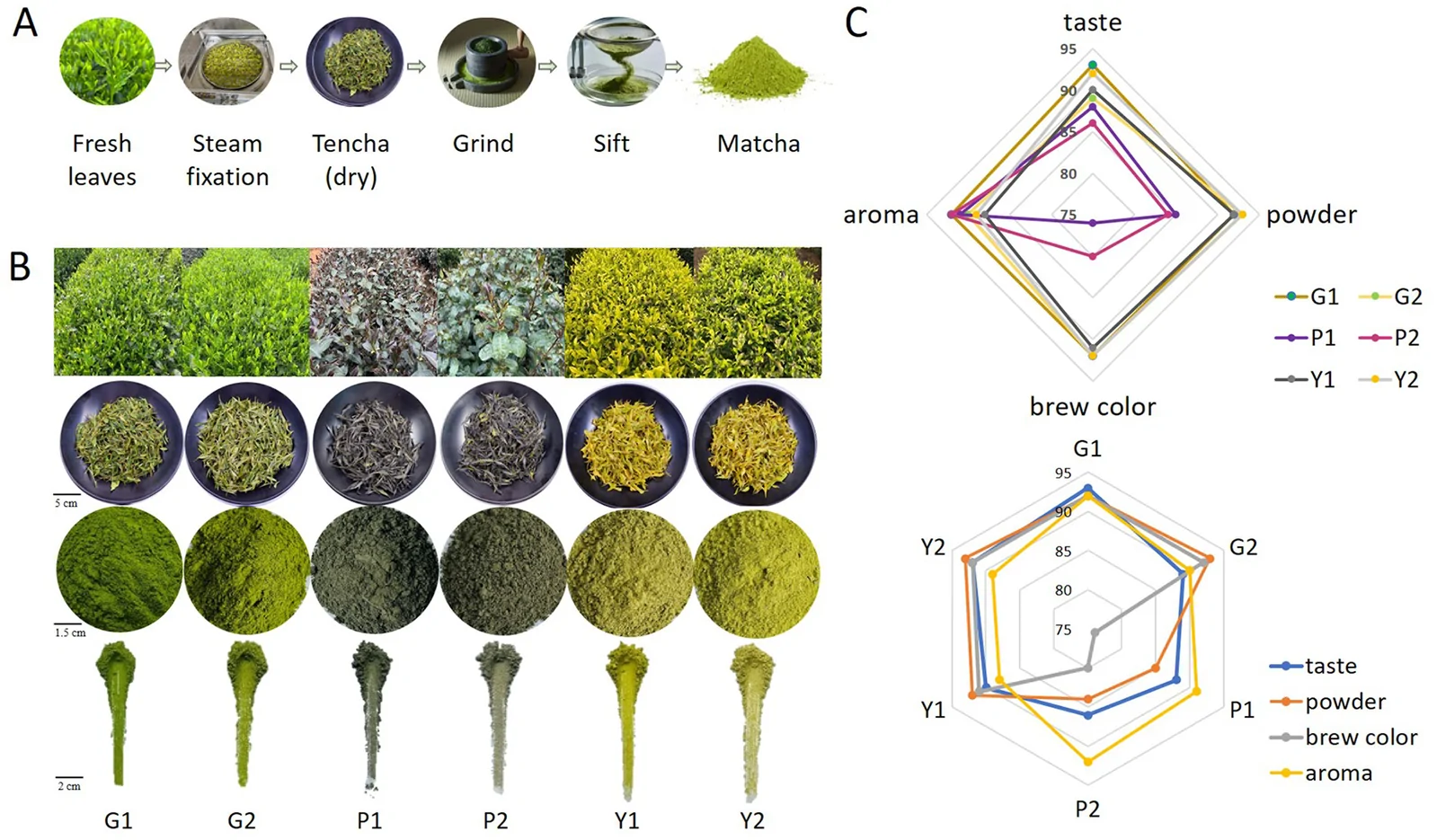
Standardized processing flowchart of matcha and sensory evaluation of matcha by leaf color. **(A)** Standardized procedures for preparing matcha from fresh tea leaves (two leaves and one bud). Tea varieties include green cultivars “*Yabukita*” (G1) and “*Pingshui Rizhu*” (G2), purple progenies of “*Zijuan*” (P1, P2), and yellow progenies of “*Baijiguan*” (Y1, Y2). **(B)** Appearance characteristics of the six matcha samples (G1, G2, P1, P2, Y1, Y2). **(C)** radar chart of scores of four core sensory indicators for six matcha samples (G1, G2, P1, P2, Y1, Y2).

The tencha was then stone-milled for 5 min and passed through a 500-μm sieve to obtain matcha powder. Each cultivar was processed independently through the complete production line under identical equipment settings, temperature profiles, processing durations, milling times, and sieving parameters to minimize operational variation among batches. Accordingly, three independently manufactured matcha batches were obtained for each cultivar and used as the three replicates (*n* = 3) in subsequent biochemical and metabolomic analyses. The resulting matcha samples were stored at −20 °C until analysis.

### 2.2 Sensory evaluation of colored-leaf matcha

Powder appearance, brew color, taste, and aroma were evaluated as the major sensory attributes of matcha quality. Six matcha samples produced from green-, purple-, and yellow-leaf tea cultivars were evaluated according to GB/T 34778-2017, Technological Specification of Matcha Sensory Evaluation. Five graduate students majoring in tea science served as the sensory panelists. Before the formal evaluation, the panelists received standardized training on matcha sensory evaluation, including the assessment of powder appearance, brew color, taste, and aroma, as well as the corresponding scoring criteria and sensory descriptors specified in the national standard. During the formal evaluation, the six samples were coded and presented in a randomized order. Each panelist independently evaluated all samples under the same evaluation conditions without discussing scores with the other panelists.

Powder appearance, brew color, taste, and aroma were assigned weights of 30%, 20%, 30%, and 20%, respectively. For each sensory attribute of each sample, the five individual scores were averaged to obtain the final score for that sensory attribute. The weighted overall sensory score was calculated according to the following (Equation 1):


$$\begin{array}{r} S_{\text{total}}=0.30\times S_{\text{appearance}}+0.20\times S_{\text{color}}+0.30\times S_{\text{taste}}\\ +0.20\times S_{\text{aroma}} \end{array}$$ (1)

where, (*S*_appearance_), (*S*_color_), (*S*_taste_), and (*S*_aroma_) represent the mean scores of the five panelists for powder appearance, brew color, taste, and aroma, respectively. The overall score was calculated by weighted summation of the four attribute scores. No formal panel-consistency statistic (e.g., intraclass correlation coefficient) was calculated, panel reliability was instead controlled through standardized training, nationally specified scoring criteria and descriptors, randomized sample presentation, and independent scoring.

### 2.3 Biochemical component analysis of colored-leaf matcha

Alkaloid and catechin components in six colored matcha samples were quantitatively detected using high-performance liquid chromatography (HPLC, Waters e 2695) following national standard GB/T 8313 “Method for the Determination of Tea Polyphenols and Catechins Content in Tea” and GB/T 8312 “Method for the Determination of Caffeine in Tea,” respectively.

Amino acid components were detected by UPLC (Waters Acquity I-Class PLUS, Milford, MA, USA). First, for amino acid extraction, 0.2 g of matcha powder was mixed with 10 mL of distilled water and incubated in a 100 °C water bath for 30 min, with gentle shaking every 10 min. The mixture was then centrifuged at 1,370 × g for 10 min and the supernatant then filtered through a 0.22 μm Millipore membrane. Subsequently, 10 μL of the membrane-filtered supernatant and 70 μL of AccQ Fluor borate buffer were added into a 2 mL centrifuge tube and vortexed thoroughly. Then, 20 μL of AccQ Fluor derivatizing reagent was added to the centrifuge tube, and immediately re-vortexed for uniform mixing. The mixture was left to stand at room temperature for 1 min, then transferred into a 2 mL autosampler vial, and then placed in a 55 °C oven and heated for 10 min. Finally, quantitative analysis was performed using UPLC. Mobile phases included: A, 100% AccQ Tag Ultra Eluent A; B, 90:10 (v/v) ultrapure water/AccQ Tag Ultra Eluent B; C, 100% ultrapure water; and D, 100% AccQ Tag Ultra Eluent.

### 2.4 UPLC MS/MS analysis of non-volatile metabolites (NVMs) in matcha samples

After vacuum freeze-drying, 50 mg of each sample was accurately weighed and mixed with 1,000 μL of extraction solvent (methanol:acetonitrile:water, 1:2:1, v/v/v). The mixture was vortexed for 30 s, homogenized with stainless-steel beads at 45 Hz for 10 min, and ultrasonically extracted in an ice-water bath for 10 min. After incubation at −20 °C for 1 h, the homogenate was centrifuged at 12,000 rpm for 15 min at 4 °C, and 300 μL of the supernatant was filtered through a 0.22-μm membrane into a 2-mL autosampler vial. For the quality control (QC) sample, 10 μL of the supernatant from each processed sample was pooled and mixed thoroughly, QC results, with Spearman correlation coefficients above 0.97 across the full dynamic range of metabolite abundances, are presented in Supplementary Table 3 and Supplementary Figure 1. Metabolite profiling was performed on a Waters ACQUITY UPLC I-Class PLUS system coupled to an AB Sciex QTRAP 6500+ mass spectrometer. Chromatographic separation was achieved on an ACQUITY HSS T3 column (2.1 × 100 mm, 1.8 μm) maintained at 50 °C, with a flow rate of 0.35 mL/min and an injection volume of 4 μL. Mobile phase A was water containing 0.1% formic acid and 5 mM ammonium acetate, and mobile phase B was acetonitrile containing 0.1% formic acid. The gradient program was 98% A from 0 to 1.5 min, 50% A at 5.0 min, 2% A from 9.0 to 10.0 min, followed by re-equilibration to 98% A at 11.0 min and maintenance until 14.0 min. Mass data were acquired in both positive and negative electrospray ionization (ESI) modes, with a source temperature of 550 °C and ion spray voltages of +5,500 V and −4,500 V, respectively. The ion source gas 1 (GS1), ion source gas 2 (GS2), and curtain gas (CUR) pressures were 50, 55, and 35 psi, respectively. Metabolites were detected by scheduled multiple reaction monitoring (MRM) with nitrogen as the collision gas, and the declustering potential (DP) and collision energy (CE) were optimized for individual MRM transitions. Metabolites were annotated using the GB-PLANT database based on precursor-ion/product-ion transitions and MS/MS information. Redundant signals from isotopes, K+, Na+, and NH4+ adducts, and fragment ions were removed, and annotations were classified into Levels 1–3 according to the available MS/MS and database-matching evidence. The KEGG, HMDB, and LIPID MAPS databases were subsequently used for metabolite classification and pathway annotation. Following this filtering and annotation procedure, 3,139 metabolites were retained for subsequent analyses. The detection workflow was based on a previously described widely targeted metabolomics method (15). Original peak areas were normalized to total peak area before downstream analyses.

### 2.5 GC–MS analysis of volatile metabolites (VMs) in matcha varieties

Matcha powder (0.8 g) was promptly transferred to a 20 mL head-space vial (Agilent, Palo Alto, CA, USA), and 4 μL of ethyl caprate (25 μg/L) was added as an internal standard. Volatile compounds were extracted by automated solid-phase microextraction (SPME) using a 1 cm, 65 μm divinylbenzene/carboxen/polydimethylsiloxane (DVB/ CAR/ PDMS) fiber (Supelco, Bellefonte, PA, USA). After incubation at 80 °C for 2 min, extraction was performed at 80 °C for 60 min, and the fiber was desorbed at 250 °C for 5 min in splitless mode. Separation was performed on an Agilent 7890B gas chromatograph coupled to a LECO Pegasus 4D time-of-flight mass spectrometer (GC-TOF-MS) using an Rxi-5Sil MS capillary column (30 m × 0.25 mm × 0.25 μm) with high-purity helium as the carrier gas at a flow rate of 3 mL/min. The injector and transfer-line temperatures were both 250 °C, and the oven temperature was programmed from 50 °C (held for 2 min) to 265 °C at 4 °C/min and held for 5 min. The ion source temperature was 220 °C, the electron energy was −70 eV, the detector voltage was 1,550 V, and the scan range was 33–500 amu with an acquisition rate of 10 spectra/s. Detection was performed as previously described with some modifications (16, 17).

VMs were characterized by matching their mass spectra against the data system library and linear retention indices (LRI). LRI values were calculated using a series of C8–C20 n-alkanes as reference standards and compared with values reported in the NIST database for non-polar GC columns. Raw data were processed using ChromaTOF software (v4.51.6, LECO Corporation, St. Joseph, MI, USA), and compounds with a minimum similarity match score of 750 were assigned names. The presence of all selected ions in the sample spectrum was verified after background subtraction. Using ethyl caprate as the internal standard (4 μL, 25 μg/L), the relative content of each metabolite was calculated according to Huang et al. (11).

### 2.6 Statistical analysis

To identify differentially accumulated metabolites (DAMs) among matcha samples, orthogonal partial least squares-discriminant analysis (OPLS-DA) was conducted for pairwise comparisons with biological replicates (*n* = 3) fitted within each group. The OPLS-DA model quality was evaluated using R^2^X, R^2^Y and Q^2^Y parameters calculated via cross-validation, and permutation testing was performed to assess the risk of overfitting (Supplementary Figure 2). DAMs were defined by Welch's *t*-test with Benjamini–Hochberg false discovery rate correction, using the criteria of *q* < 0.05, variable importance in projection (VIP) score ≥ 1, and |fold change| ≥ 2. The resulting *q*-values are reported in Supplementary Table 4. Metabolites with KEGG pathway annotation were retained for downstream enrichment analysis. To visualize common and unique components, heatmap analysis and principal component analysis (PCA) were conducted via an online platform (https://cloud.metware.cn/). The Kyoto Encyclopedia of Genes and Genomes (KEGG) database was used to annotate and classify DAM functions. Graphical representations were generated using Origin 2018. For multiple comparisons, one-way analysis of variance (ANOVA) followed by Tukey's honestly significant difference (HSD) *post hoc* test was used to determine significant differences (*p* < 0.05). The values are presented as means ± standard deviations.

## 3 Results and discussion

### 3.1 Sensory characteristics of matcha from leaf-color cultivars

Appearance, taste, and aroma are primary determinants of matcha quality. Sensory evaluation revealed clear differences among matcha produced from each cultivar (Figure 1B). Green matcha, with G1 as the quality reference, had hallmark characteristics of premium matcha, including a bright green appearance, clear green infusion, fresh taste, and pronounced seaweed-like aroma. It achieved consistently high and balanced scores across all sensory attributes (total score 92.3 calculated by Equation 1), reflecting strong sensory harmony. Yellow matcha had yellow- to apricot-colored leaves, fine powder texture, and high appearance scores (92-93). Its taste was dominated by umami, while the aroma featured mild glutinous notes. Although its infusion color differed from that of green matcha, its overall sensory score (90.4–91.7) was comparable, indicating strong potential as a high-quality product with a distinct sensory profile. Purple matcha deviated from conventional matcha standards in appearance and infusion color, resulting in lower infusion color scores. While it was also slightly bitter and astringent, it had outstanding aroma performance, with scores comparable to those of green matcha. Its fresh, holly-like aroma suggests potential for aroma-based product differentiation (Figure 1C; Supplementary Table 2).

### 3.2 Mechanistic links between major biochemical components and matcha quality

Catechins, particularly epigallocatechin gallate (EGCG), are the dominant polyphenols in tea and serve as major contributors to antioxidant capacity and astringency (4). Amino acids, with theanine as the key representative, primarily determine umami and a sweet-mellow taste while modulating bitterness and astringency through taste antagonism. Alkaloids, mainly caffeine, contribute bitterness and act synergistically with catechins to intensify astringency. The sensory characteristics of matcha, therefore, emerge from the dynamic balance among these compound classes rather than from their absolute concentrations alone (Table 1).

**Table 1:** Major biochemical components of matcha by leaf color.

Class	Component	G1 (%)	G2 (%)	P1 (%)	P2 (%)	Y1 (%)	Y2 (%)
Amino acid	Thea	1.35 ± 0.01 e	2.60 ± 0.08 d	2.72 ± 0.03 c	2.61 ± 0.05 d	3.15 ± 0.09 a	3.02 ± 0.02 b
	Gln	0.07 ± 0.00 e	0.12 ± 0.00 d	0.21 ± 0.01 c	0.11 ± 0.03 d	0.29 ± 0.01 a	0.23 ± 0.08 b
	Arg	0.18 ± 0.04 b	0.35 ± 0.01 a	0.05 ± 0.00 d	0.02 ± 0.01 e	0.11 ± 0.00 c	0.18 ± 0.00 b
	Glu	0.41 ± 0.01 b	0.51 ± 0.01 a	0.24 ± 0.01 cd	0.22 ± 0.01 d	0.27 ± 0.01 c	0.40 ± 0.02 b
	Asp	0.38 ± 0.03 a	0.16 ± 0.00 d	0.20 ± 0.01 c	0.25 ± 0.01 b	0.21 ± 0.05 c	0.25 ± 0.02 b
	Ala	0.05 ± 0.01 a	0.04 ± 0.00 b	0.03 ± 0.00 c	0.04 ± 0.00 b	0.04 ± 0.00 b	0.04 ± 0.00 b
	Ser	0.05 ± 0.00 c	0.05 ± 0.00 c	0.07 ± 0.00 a	0.07 ± 0.00 a	0.06 ± 0.00 b	0.07 ± 0.00 a
	Thr	0.05 ± 0.00 b	0.04 ± 0.00 c	0.06 ± 0.00 a	0.04 ± 0.00 c	0.05 ± 0.00 b	0.05 ± 0.00 b
Alkaloid	TB	0.10 ± 0.00 f	0.23 ± 0.02 d	0.73 ± 0.04 a	0.69 ± 0.01 b	0.56 ± 0.02 c	0.19 ± 0.01 e
	CAF	4.44 ± 0.09 d	5.18 ± 0.03 a	5.09 ± 0.02 b	4.45 ± 0.06 d	4.82 ± 0.07 c	3.22 ± 0.01 e
Catechin	EGC	2.14 ± 0.04 e	3.48 ± 0.10 d	4.01 ± 0.02 c	1.53 ± 0.03 f	4.13 ± 0.10 b	5.39 ± 0.11 a
	EC	0.34 ± 0.01 e	0.53 ± 0.05 c	0.46 ± 0.00 d	0.32 ± 0.01 e	0.92 ± 0.01 b	1.35 ± 0.02 a
	EGCG	10.38 ± 0.09 d	14.20 ± 0.15 c	16.25 ± 0.18 a	15.40 ± 0.30 b	13.93 ± 0.30 c	15.92 ± 0.39 ab
	ECG	1.63 ± 0.01 e	2.39 ± 0.02 d	2.68 ± 0.02 c	3.40 ± 0.07 b	4.47 ± 0.01 a	2.81 ± 0.03 c

Letters (a-f) indicate significant differences among groups (*p* < 0.05, ANOVA).

Quantitative analysis revealed pronounced cultivar-dependent differences in amino acid composition. Yellow matcha had the highest theanine and glutamine levels, compounds known to enhance umami perception and mouthfeel. Green matcha had higher arginine and aspartic acid levels, both implicated in enhancing freshness and umami intensity (18). The coordinated enrichment of these amino acids likely underpins the characteristic fresh and savory taste of green matcha. Elevated levels of serine and threonine in purple and yellow matcha may contribute to taste complexity and metabolic flux toward aroma-related pathways.

Catechin and alkaloid profiles differed markedly among matcha types, providing a biochemical basis for differences in bitterness and astringency. Yellow matcha had the highest total catechin content, and purple matcha had the highest EGC, caffeine, and total alkaloid levels. Because EGCG and caffeine act synergistically to enhance bitterness and astringency, their co-enrichment in purple matcha likely explains its perceptible post-bitterness despite relatively high theanine content. In contrast, green matcha had substantially lower total catechins, EGCG, and caffeine, which likely attenuated bitterness and astringency, allowing umami-related amino acids to dominate sensory perception. The high epicatechin gallate (ECG) content in yellow matcha, together with elevated theanine and glutamine levels, suggests a distinct taste-regulation mechanism. ECG contributes moderate astringency but exhibits a smoother sensory profile than EGCG, while amino acids buffer astringent perception. Moreover, glutamine may participate in Maillard-related reactions during processing, contributing to the glutinous aroma notes detected in sensory evaluation. Collectively, these results demonstrate that matcha quality is governed by the coordinated regulation of amino acid, polyphenol, and alkaloid interactions rather than by any single compound class. Cultivar-dependent metabolic reprogramming alters the relative proportions of these components, affecting taste, aroma, and overall sensory quality through well-defined biochemical mechanisms.

### 3.3 Effects of shading on the chemical quality of colored-leaf matcha

Matcha quality is influenced by both pre-harvest cultivation practices and post-harvest processing. This is particularly important for non-green-leaf cultivars, in which shading may alter leaf-color-associated pigments and taste-related metabolites, while thermal processing may further affect pigment stability. Pre-harvest shading is widely used in matcha production because reduced light availability can enhance chlorophyll accumulation and alter the balance of amino acids and polyphenols, thereby contributing to umami-rich and less astringent sensory characteristics (19). However, these responses are strongly dependent on cultivar and shading conditions. In particular, light-sensitive yellow-leaf cultivars, such as “Huangjinya” and “Baijiguan,” can gradually re-green under reduced light, accompanied by increased chlorophyll accumulation and changes in other metabolites (20, 21). Thus, shading regimes that benefit conventional green-leaf matcha may not produce the same phenotypic or sensory effects in yellow-leaf cultivars. In the present study, a relatively short shading period, from the fish-leaf stage to the one bud and two leaves stage, was applied to minimize changes in characteristic leaf coloration. Previous studies have similarly shown that the effects of shading duration and intensity on matcha quality are cultivar-dependent, and prolonged shading does not necessarily further improve quality (19). Therefore, the optimal shading regimes for non-green-leaf cultivars warrant further investigation.

### 3.4 NVMs profiles of matcha by leaf color

#### 3.4.1 Global characterization of NVMs

Tea flavor quality is collectively determined by the type, content, and proportion of NVMs and VMs (22). NVMs directly influence the color and mouthfeel of tea infusions, with pigment substances such as chlorophyll, carotenoids, and anthocyanins dominating the appearance of tea leaves and tea infusion color. Widely targeted metabolomics identified 3139 NVMs across all samples, encompassing amino acids, flavonoids, terpenoids, carbohydrates, alkaloids, organic acids, polyphenols, lipids, nucleotides, and other compound classes (Figure 2A; Supplementary Table 3). Purple-leaf matcha had higher total flavonoid contents, and yellow-leaf matcha had higher total amino acid levels (Figure 2B), suggesting distinct metabolic allocation patterns associated with leaf color. PCA revealed clear separation among the six samples (Figure 2C). The first principal component (PC1, 28.3% variance) distinguished green (G1, G2) from purple matcha (P1, P2), while the second (PC2, 21.3% variance) separated yellow matcha (Y1, Y2). Samples clustered tightly within leaf-color groups, indicating high metabolic similarity within and pronounced metabolic divergence among them. These results confirm that the NVM profile of matcha differs markedly among cultivars representing different leaf-color categories.

**Figure 2:**
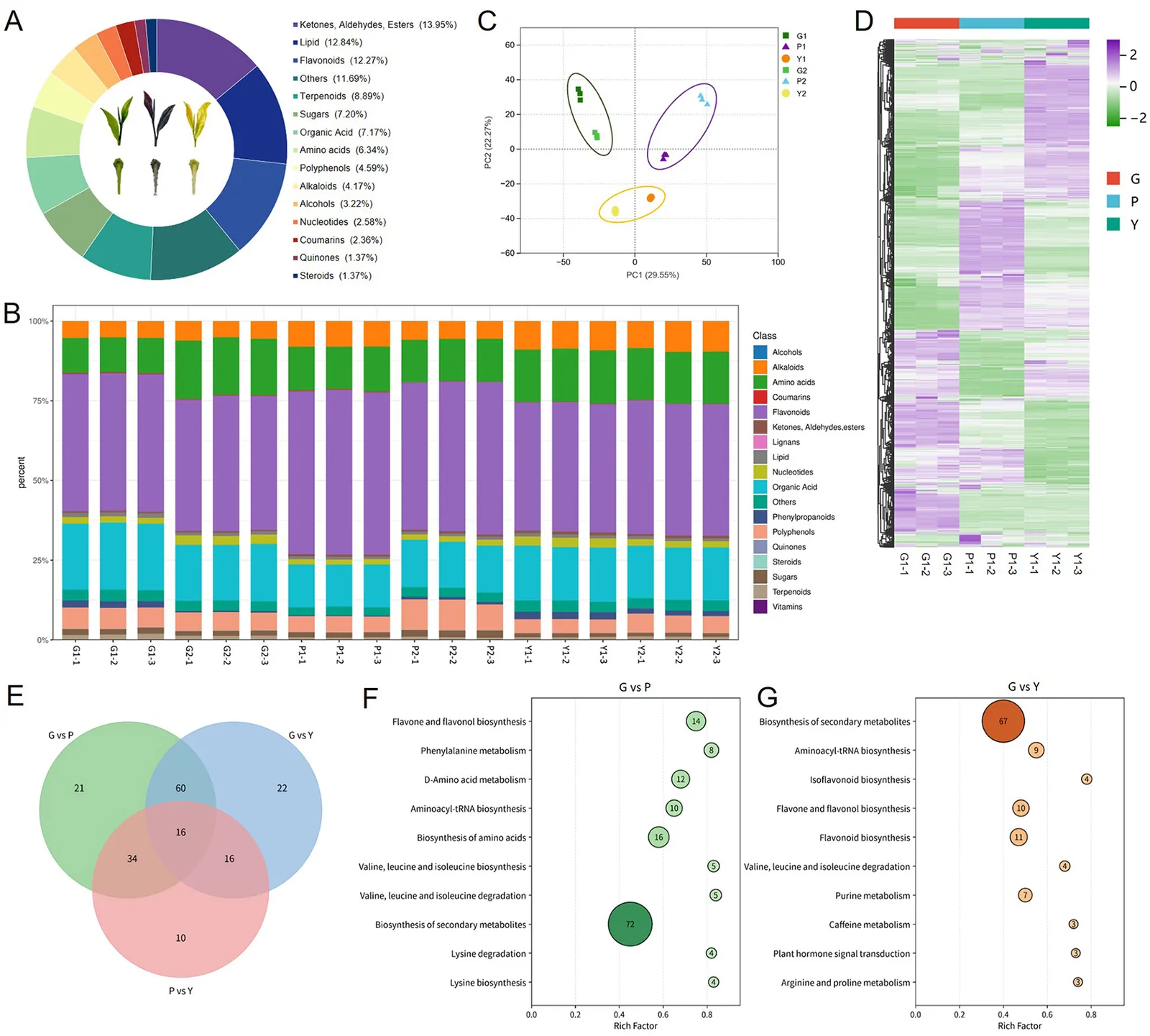
Global characteristics and differential analysis of NVMs in matcha by leaf color. **(A)** Pie chart of NVM category distribution. **(B)** bar chart of NVM category contents. **(C)** principal component analysis of NVMs (Hotelling's T^2^ 95% confidence ellipses were applied to show the within-group variation.). **(D)** hierarchical clustering heatmap of NVMs (Each column represents an individual biological replicate, the hierarchical clustering was performed based on Euclidean distance and Ward's linkage method). **(E)** Venn diagram of differential metabolites among groups and KEGG enrichment plot of differentially accumulated metabolites between green vs. purple **(F)** and yellow matcha **(G)**.

#### 3.4.2 Differential NVMs among matcha types

To highlight the characteristic metabolic divergence among cultivars representing different leaf-color categories while minimizing redundant intragroup cultivar complexity, one representative cultivar per category was predefined before data analysis based on the most prominent leaf pigmentation and typical agronomic attributes, and was then used for deep comparative dissection: G1 (standard matcha-producing cultivar), P1 (distinct purple), and Y1 (characteristic albino). PCA confirmed that cultivars clustered by leaf-color phenotype (Figure 2C), supporting the use of a single representative cultivar from each color category. Because each leaf-color group is represented by a single cultivar in this focused comparison, cultivar identity and leaf-color category cannot be statistically distinguished, the differential analyses below should therefore be interpreted as an exploratory comparison of three selected cultivars representing the three leaf-color groups, rather than as a general assessment of leaf-color effects independent of cultivar background. Hierarchical clustering of these typical cultivars revealed distinct metabolite accumulation patterns among green, purple, and yellow matcha (Figure 2D). Differential metabolite analysis identified 131 DAMs between green and purple teas, 76 between purple and yellow teas, and 114 between green and yellow teas (Figure 2E; Supplementary Table 4). Sixteen DAMs were shared across all three pairwise comparisons, including representative flavonoids, amino acids, organic acids, phenylpropanoids, and alkaloids. KEGG enrichment analysis revealed that phenylalanine metabolism, flavone and flavonol biosynthesis, D-amino acid metabolism, branched-chain amino acid metabolism, and aminoacyl-tRNA biosynthesis were prominently enriched in the green vs. purple comparison (Figure 2F). Phenylalanine is the primary precursor for the phenylpropanoid and flavonoid pathways, its enrichment, together with flavonoid metabolism, is closely associated with purple-leaf formation, as anthocyanins and other flavonoids preferentially accumulate in purple tea leaves (23, 24). This was consistent with the higher anthocyanin and flavonol levels and stronger bitterness/astringency observed in purple matcha, as flavonoids are important contributors to tea astringency (25). The enrichment of D-amino acid metabolism and aminoacyl-tRNA biosynthesis further indicated changes in amino-acid-related metabolism, consistent with cultivar-dependent differences in free amino acids associated with umami and mellow taste (26, 27).

In the green vs. yellow comparison (Figure 2G), flavonoid-related pathways (isoflavonoid biosynthesis, flavonoid biosynthesis, and flavone and flavonol biosynthesis), amino-acid metabolism (aminoacyl-tRNA biosynthesis, arginine and proline metabolism), and caffeine metabolism showed prominent enrichment. The enrichment of aminoacyl-tRNA and amino-acid metabolic pathways was consistent with the altered amino-acid metabolism and higher amino-acid abundance associated with the umami-dominant and mellow sensory profile of yellow matcha. The enrichment of flavonoid-related pathways was consistent with the higher flavonol glycoside levels observed in yellow matcha, which contribute to its yellowish-green infusion color and moderate bitterness. Overall, these results suggest that the metabolic differentiation among matcha from the studied leaf-color cultivar groups is accompanied by coordinated changes in flavonoid and amino-acid-related metabolism.

Anthocyanins are characteristic metabolites of purple-leaf tea and contribute to its distinctive coloration and antioxidant properties. In our metabolomic analysis, several anthocyanins, including cyanidin-3-O-galactoside, cyanidin 3-rutinoside, and kuromanin, showed higher signal intensities in purple matcha than in green- and yellow-leaf samples. Consistently, purple-leaf tea has been reported to contain substantially higher levels of anthocyanins, particularly delphinidin- and cyanidin-glycosides, than green-leaf tea (28). Purple matcha was also enriched in anthocyanins and flavonols, including cyanidin 3-rutinoside, rutin, kuromanin, baimaside, pelargonidin chloride, nicotiflorin, and rhoifolin, which may contribute to its purple color and stronger bitterness and astringency. Anthocyanins are sensitive to thermal processing, although their stability depends on their composition and interactions with other tea constituents. For instance, an anthocyanin-rich extract from “Zijuan” showed reduced stability with increasing temperature (29), while gallic acid and EGCG had differential effects on anthocyanin stability in purple-tea infusions (28). Thus, high-temperature processing may affect anthocyanin retention in purple matcha, but the extent and direction of these changes likely depend on the anthocyanin profile and processing matrix. It should also be noted that anthocyanins were not quantified in the same biological material before and after matcha processing, therefore, the magnitude of anthocyanin degradation during matcha manufacture cannot be quantitatively established from the present data. Purple matcha also showed elevated levels of L-valine, a bitter amino acid that may contribute to taste complexity when balanced with umami amino acids (22). L-Valine can participate in Maillard reactions with reducing sugars during high-temperature processing and contribute to the formation of pyrazine compounds associated with roasted and nutty aromas in tea products (30). In addition, two terpenoids, (R)-(+)-citronellal and sclareol, accumulated at higher levels in purple matcha. (R)-(+)-Citronellal is a monoterpene aldehyde associated with citrus and fresh floral aromas (31–33), whereas sclareol is characterized by a musky, amber-like aroma and is known for its strong and persistent fragrance properties (34, 35). The accumulation of these volatile compounds may therefore contribute to the distinctive aroma profile of purple matcha.

Yellow matcha had high accumulations of amino acids such as L-lysine, L-glutamine, D-phenylalanine, L-phenylalanine, L-histidine, L-leucine, L-isoleucine, L-proline, and D-proline, which contributed to its umami-sweet taste. It also had relatively high levels of flavonols (isoquercitrin, quercitrin, and kaempferol-3-O-galactoside). Flavonol glycosides (e.g., quercetin glycosides, kaempferol glycosides) deepen the yellow color of tea infusions and have extremely low taste thresholds, which enhance bitterness and astringency (36). This partly explains our sensory evaluation results, where the taste of yellow matcha was slightly more bitter than that of green matcha, and it exhibited a yellowish-green infusion color.

### 3.5 Characterization of VMs in matcha varieties

#### 3.5.1 VMs identified from matcha varieties

A total of 67 VMs were identified across the three matcha types, including terpenes, esters, alcohols, ketones, and other compound classes (Supplementary Table 5). Of these, terpenes were the most abundant category of detected volatile compounds, accounting for 25.37% (Figure 3A). This class of compounds typically imparts diverse aroma characteristics, such as floral (37, 38) and woody and citrus (39) notes. The next most abundant categories were esters (16.42%), alcohols (11.94%), and ketones (11.94%). Esters such as propyl phenylacetate and 2-phenylethyl ester often contribute fruity and sweet aromas (40), alcohols provide green grassy and sweet notes, while ketones generally offer caramel and nutty aromas (41). Together, these four categories constitute the major portion of the volatile profile detected in matcha. Other detected categories included heterocyclics (8.96%) and aldehydes (5.97%), which are typically associated with roasted and fatty or green grassy notes, respectively. What remained comprised trace components such as alkanes, carboxylic acids, and aromatic hydrocarbons, which may play roles in supporting and harmonizing the overall aroma. These results indicate that the unique volatile profile of matcha is shaped by a complex system dominated by terpenes, esters, and alcohols, with the synergistic effect of various other classes of volatile substances.

**Figure 3:**
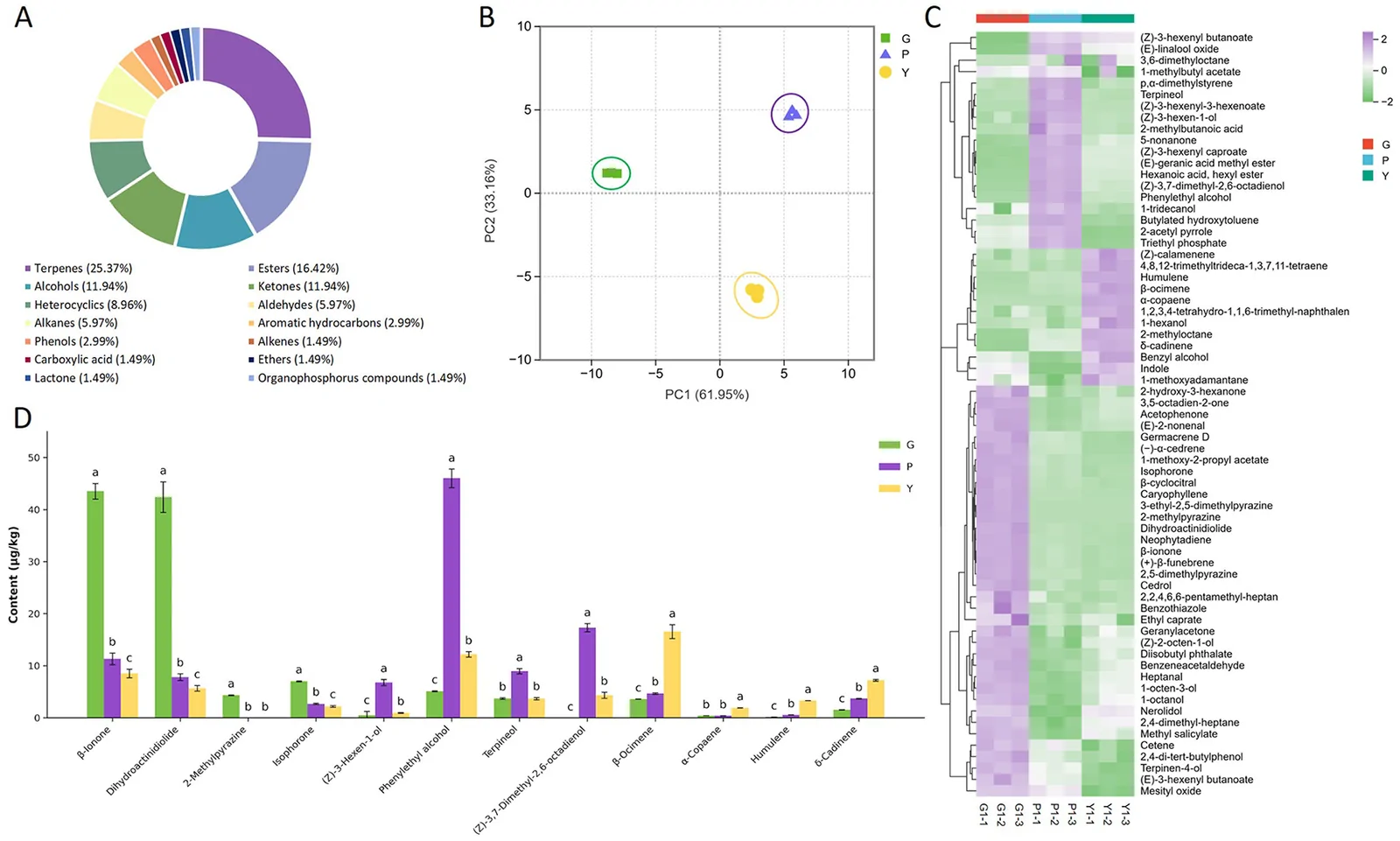
Composition of volatile metabolites and analysis of putative aroma-related volatiles in matcha by leaf color. **(A)** Pie chart of volatile metabolites category proportion. **(B)** principal component analysis of volatile metabolites. **(C)** hierarchical clustering heatmap of volatile metabolites (Each column represents an individual biological replicate, the hierarchical clustering was performed based on Euclidean distance and Ward's linkage method) and **(D)** bar chart of the contents of putative aroma-related volatiles.

#### 3.5.2 Putative aroma-related volatiles in matcha varieties

PCA revealed the VMs of each matcha type to distinctly separate (Figure 3B). Heatmap analysis revealed significant differences in their volatile substance contents. Green matcha contained the most abundant volatile substances, followed by purple, then yellow matcha (Figure 3C).

The most representative aroma of green matcha is the seaweed-like note, which results from the synergistic effect of multiple volatile components. This characteristic aroma is likely derived from carotenoid degradation products in tea leaves (e.g., dihydroactinidiolide and β-ionone). Dihydroactinidiolide exhibits intense herbal, woody, and seaweed-like aromas with a subtle sweet undertone, and β-ionone imparts violet floral and berry-like fruity notes. Consistent with our results, Zhang (42) reported both α-ionone and β-ionone to be key components of the seaweed-like aroma in Shandong matcha. Additionally, Huang (11) performed a metabolomic analysis of matcha made from “*Okumidori*,” “*Longjing 43*,” “*Zhongcha 108*,” and “*Echa 1*” green tea cultivars and concluded that “aldehydes account for the most vital contribution in the aroma quality formation of matcha” (11). We report the content of aldehydes (e.g., heptanal, benzeneacetaldehyde, (E)-2-nonenal, and β-cyclocitral) in green matcha to be significantly higher than those in either other matcha type. Furthermore, ketones (including geranylacetone, acetophenone, and isophorone), terpenes (such as caryophyllene), and heterocyclics may also play important roles in shaping the overall aroma profile of green matcha, collectively contributing to its complex and layered aroma characteristics.

The volatile substances detected in purple matcha were dominated by floral-fruity, sweet, and fresh notes, with alcohols, esters, and terpenes potentially contributing to these aroma characteristics. Alcohols are important to the fresh aroma of matcha (43), and phenylethanol, geraniol, (Z)-3-hexenol drive *Qianmei 502* black tea's sweet floral aroma (44). We report (Z)-3-hexen-1-ol, terpineol, phenylethyl alcohol, and (Z)-3,7-dimethyl-2,6-octadienol to be abundant in purple matcha, which may be associated with its fresh and floral notes. The aroma composition of yellow matcha was dominated by terpenes and alcohols. Various substances (e.g., β-ocimene, α-copaene, δ-cadinene, and humulene and related substances) may contribute to fruity and herbal notes (Figure 3D), and trace heterocyclic compounds (e.g., pyrrole, indole) may be associated with nutty and roasted notes. Combined with the results of previous research, yellow matcha had the highest amino acid content, these amino acids inherently possess a sweet taste and undergo complex interactions such as the Maillard reaction with carbohydrates, endowing the tea infusion with a smooth, rice soup-like mellow mouthfeel. This is highly consistent with our sensory evaluation results.

## 4 Conclusion

We integrate sensory evaluation and multi-omics analyses to characterize quality differentiation among matcha produced from cultivars representing different leaf-color categories. Green matcha served as the quality benchmark with umami-rich freshness and balanced aroma, yellow matcha had comparable quality characterized by pronounced umami and a distinctive glutinous aroma, and purple matcha had unique aroma attributes despite deviations in appearance and taste. Metabolomic analyses revealed these sensory differences to arise from coordinated regulation of amino acids, catechins, flavonols, and detected volatile compounds, with flavonoid biosynthesis and amino acid metabolism playing central roles. Overall, results and their interpretation provide a concise biochemical framework for cultivar selection and the development of differentiated matcha products based on leaf-color traits (Figure 4).

**Figure 4:**
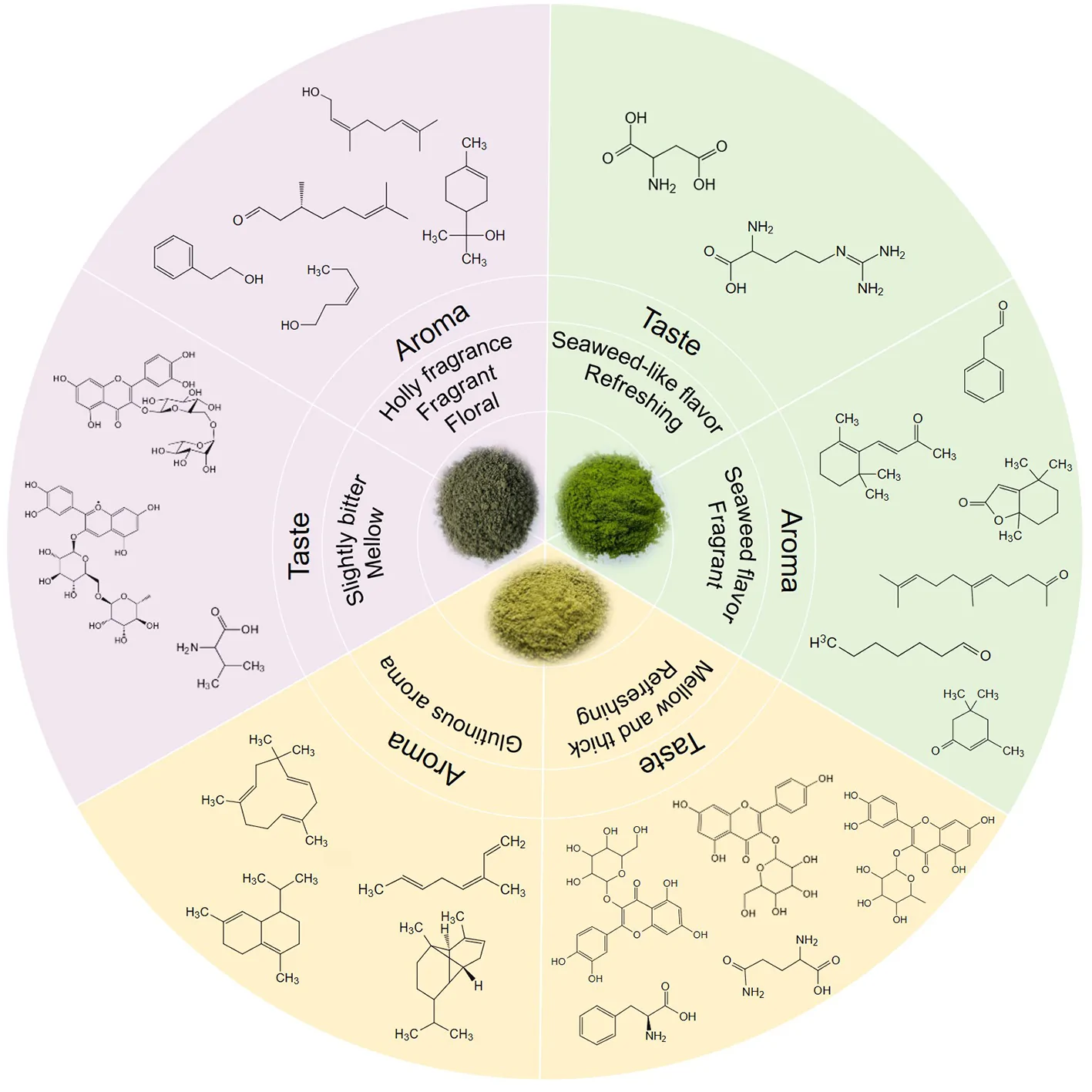
Diagram of the biochemical mechanisms underlying flavor differences among matcha produced from cultivars with different leaf colors. This figure integrates multi-omics data from sensory evaluation, NVMs, and VMs to construct a core biochemical mechanism framework for flavor formation in matcha from cultivars with different leaf colors. The circle is divided into three sectors according to matcha color. The right side of sectors marks the aroma-related module, presenting the chemical structures of putative aroma-related volatiles and their corresponding flavor characteristics. The left side marks the taste-related module, showing the chemical structures of key taste substances and their corresponding taste characteristics.

## Data Availability

The original contributions presented in the study are included in the article/Supplementary material, further inquiries can be directed to the corresponding authors.
