# Supplementary material for: Metabolic characteristics and chemical basis of matcha from cultivars with different leaf colors
Source: Front Nutr. 2026 Oct 19;13:1951952. doi: 10.3389/fnut.2026.1951952 (PMC13724418; doi:10.3389/fnut.2026.1951952)
Supplement: Supplementary file 1 [file Supplementary_file_1.docx]

Supplementary Figure 1. Assessment of instrument stability and analytical reproducibility across pooled quality control (QC) samples. Pairwise scatter plots (lower left) and Spearman correlation coefficients (upper right) among the four QC runs injected across the sample batch.

The high correlation coefficients (r > 0.97) across the full dynamic range of metabolite abundances confirm high operational reproducibility and analytical stability of the UPLC-MS/MS platform throughout the sequence.

Supplementary Figure 2. OPLS-DA model validation for pairwise comparisons among matcha samples.

a, b: G1 (A-1, A-2, A-3) vs. P1 (C-1, C-2, C-3);

c, d: G1 (A-1, A-2, A-3) vs. Y1 (F-1, F-2, F-3);

e, f: P1 (C-1, C-2, C-3) vs. Y1 (F-1, F-2, F-3).

OPLS-DA score plots with R²X, R²Y, and Q²Y values (a, c, e) ;permutation test results. Q²Y intercepts below zero support model reliability and indicate no apparent overfitting (b, d, f)
